# Chronic Over-Expression of Heat Shock Protein 27 Attenuates Atherogenesis and Enhances Plaque Remodeling: A Combined Histological and Mechanical Assessment of Aortic Lesions

**DOI:** 10.1371/journal.pone.0055867

**Published:** 2013-02-07

**Authors:** Charles M. Cuerrier, Yong-Xiang Chen, Dominique Tremblay, Katey Rayner, Melissa McNulty, XiaoLing Zhao, Christopher R. J. Kennedy, Jacqueline de BelleRoche, Andrew E. Pelling, Edward R. O'Brien

**Affiliations:** 1 University of Ottawa Heart Institute, Ottawa, Canada; 2 Centre for Interdisciplinary NanoPhysics, Department of Physics, University of Ottawa, Ottawa, Canada; 3 Departments of Medicine and Cellular and Molecular Medicine, University of Ottawa, Ottawa, Canada; 4 Imperial College, London, United Kingdom; 5 Department of Biology, University of Ottawa, Ottawa, Canada; 6 Institute for Science, Society and Policy, University of Ottawa, Ottawa, Canada; 7 Libin Cardiovascular Institute of Alberta, Calgary, Canada; University of Illinois at Chicago, United States of America

## Abstract

**Aims:**

Expression of Heat Shock Protein-27 (HSP27) is reduced in human coronary atherosclerosis. Over-expression of HSP27 is protective against the early formation of lesions in atherosclerosis-prone apoE^−/−^ mice (apoE^−/−^HSP27^o/e^) - however, only in females. We now seek to determine if chronic HSP27 over-expression is protective in a model of advanced atherosclerosis in both male and female apoE^−/−^ mice.

**Methods and Results:**

After 12 weeks on a high fat diet, serum HSP27 levels rose more than 16-fold in male and female apoE^−/−^HSP27^o/e^ mice, although females had higher levels than males. Relative to apoE^−/−^ mice, female apoE^−/−^HSP27^o/e^ mice showed reductions in aortic lesion area of 35% for *en face* and 30% for cross-sectional sinus tissue sections – with the same parameters reduced by 21% and 24% in male cohorts; respectively. Aortic plaques from apoE^−/−^HSP27^o/e^ mice showed almost 50% reductions in the area occupied by cholesterol clefts and free cholesterol, with fewer macrophages and reduced apoptosis but greater intimal smooth muscle cell and collagen content. The analysis of the aortic mechanical properties showed increased vessel stiffness in apoE^−/−^HSP27^o/e^ mice (41% in female, 34% in male) compare to apoE^−/−^ counterparts.

**Conclusions:**

Chronic over-expression of HSP27 is atheroprotective in both sexes and coincides with reductions in lesion cholesterol accumulation as well as favorable plaque remodeling. These data provide new clues as to how HSP27 may improve not only the composition of atherosclerotic lesions but potentially their stability and resilience to plaque rupture.

## Introduction

Heat Shock Protein 27 (HSP27) is a member of the small heat shock protein family and is involved in a wide variety of cellular processes, both physiological and pathological. Originally described as an intracellular chaperone, HSP27 is capable of binding and stabilizing the actin cytoskeleton in response to stress as well as preventing downstream caspase activation [1]. More contemporary studies now show that HSP27 and other (unrelated) heat shock proteins may have an extracellular role and signal via discrete receptors [2]. Indeed, we recently demonstrated that, via NF-κB activation, extracellular HSP27 regulates the expression of a number of genes, including several of direct relevance to regulation of vessel wall inflammation [3].

The expression of HSP27 in atherosclerotic plaques diminishes with progression of the stage of the pathology [4], [5]. Moreover, serum levels of HSP27 are reduced in patients with carotid artery disease [5] yet are increased in acute coronary syndromes [6]. Decreased HSP27 expression may be an important factor in the development of atherosclerosis; hence to address this question we performed acute studies of atherosclerosis-prone apoE^−/−^ mice that over-express HSP27 (i.e., apoE^−/−^HSP27^o/e^) [7]. After four weeks of ingesting a high fat diet (HFD) there was approximately a 35% reduction in the extent of aortic atherosclerosis in apoE^−/−^HSP27^o/e^ relative to apoE^−/−^ mice – but only in females. There are several important findings from our original atherogenesis studies involving apoE^−/−^HSP27^o/e^ mice. First, while baseline serum levels of HSP27 are virtually undetectable in all mice (prior to ingesting a HFD), there was a >10-fold increase in serum HSP27 levels in female apoE^−/−^HSP27^o/e^ mice after 4 weeks of HFD but practically no change in male counterparts. Second, in female apoE^−/−^HSP27^o/e^ mice there was an inverse correlation (r^2^ = 0.78) between HSP27 levels and extent of atherosclerotic burden. Third, sex-specific *in vivo* atheroprotection coincided with our *in vitro* demonstration that the release of HSP27 from macrophages occurs in response to estrogens. Finally, we subsequently demonstrated that ovariectomized apoE^−/−^HSP27^o/e^ mice do not show elevated serum HSP27 levels or atheroprotection until they are given estrogen replacement therapy [8]. However, it is important to note that, in addition to estrogens promoting the release of HSP27 into the extracellular space, we previously observed *in vitro* that acLDL did so as well. Hence, the question remains – could chronic exposure to an atherogenic diet that is enriched in cholesterol promote the release of HSP27 into the serum (and therefore atheroprotection) in male mice – but perhaps requires higher serum levels of cholesterol and/or more chronic exposure (>4 weeks) to be detectable?

In anticipation of future studies designed to explore the potential of HSP27 as a novel therapeutic agent (e.g., recombinant HSP27 protein), we sought to determine if over-expression of HSP27 is chronically atheroprotective and alters not only plaque burden but also morphology as reflected by a unique assessment of both the histology and mechanical properties of aortic lesions.

Briefly, we now demonstrate that chronic HSP27 over-expression attenuates atherogenesis in both males and females and is accompanied not only by reductions in lesions size but also arterial wall cholesterol content, macrophages accumulation and apoptosis. Moreover, we note that the aortae of these mice show increased intimal smooth muscle cell and collagen content as well as increased vessel stiffness. This study provides new evidence that the atheroprotective effect of HSP27 results not only in reduced plaque burden but also an enhanced histo-mechanical profile of lesions.

## Methods

### Murine atherosclerosis model

All experimental procedures involving laboratory animals were approved by the Animal Care and Use Committee of the University of Ottawa and complied with the United States National Institute of Health Guide for the Care and Use of Laboratory Animals. Transgenic mice with a C57BL10/CBA background and over-expressing human heat shock protein 27 (HSP27^o/e^) under the control of a chicken β-actin promoter and a CMV enhancer element were provided by Imperial College London. ApoE^−/−^ mice with the C57BL/6 genetic background were purchased from the Jackson Laboratory (Bar Harbor, Maine). HSP27^o/e^ females were crossed with apoE^−/−^ males to generate apoE^+/−^HSP27^o/e^ mice, which were then crossed with apoE^−/−^ mice to generate apoE^−/−^HSP27^o/e^ and apoE^−/−^ littermates [7], [9]. Each study cohort consisted of at least six mice.

**Figure 1:**
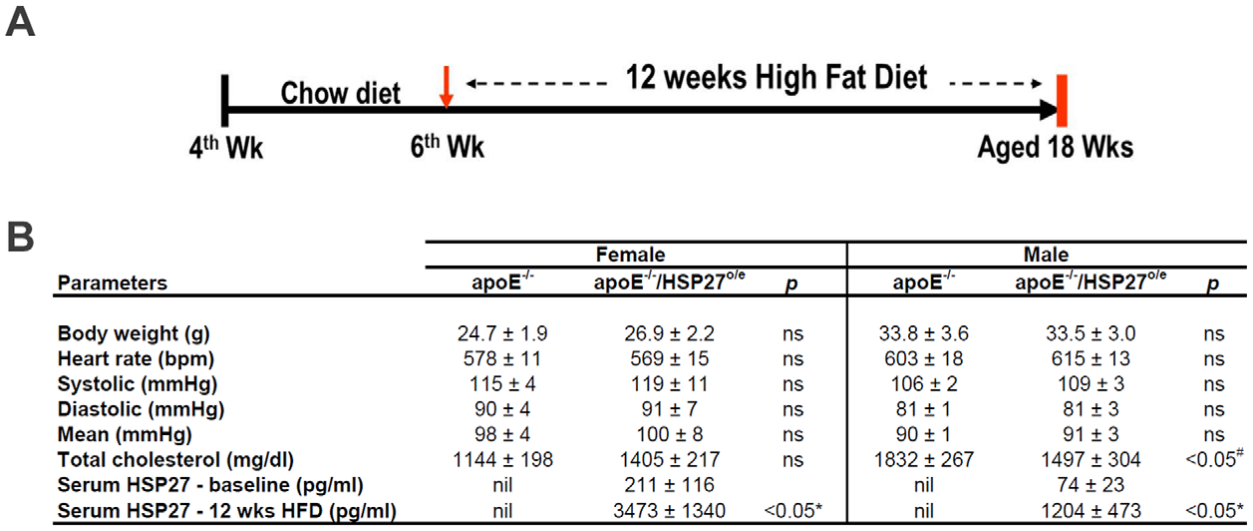
Experimental timeline and physiological parameters. (**A**) Schematic overview of chronic HSP27 over-expression atheroprotection experiment in apoE^−/−^ mice. (**B**) Table profiling the physiological parameters and the 16-fold increase in serum HSP27 levels in male and female mice maintained on a HFD for 12 weeks.

Mice were fed a normal chow diet until 6 weeks of age and thereafter received a high-fat diet (HFD, 1.25% cholesterol, 15.8% fat; Harlan Teklad, Madison, WI) for 12 weeks until euthanasia when blood samples were collected (**Figure 1A**). Depending on the nature of the intended experiment, the aortae were harvested as follows. For histological evaluations, animals were anaesthetized under isoflurane, and whole blood was collected via cardiac puncture before systemically perfusing the mice via the left ventricle with phosphate-buffered saline (PBS) followed by 4% paraformaldehyde (PFA) in PBS, before the heart and aorta were removed and immersed in 4% PFA/PBS at 4°C overnight. To quantify the aortic mechanical properties, the heart and aorta were immediately removed after euthanasia and immersed in Krebs solution (118.1 mM NaCl, 11.1 mM D-glucose, 25 mM NaHCO_3_, 4.7 mM KCl, 1.2 mM MgSO_4_•7H_2_O, 1.2 mM KH_2_PO_4_, 2.5 mM CaCl_2_•6H_2_O, pH: 7.4) at 37°C and immediately processed.

### Hemodynamic parameters

Hemodynamic parameters (heart rate, systolic and diastolic blood pressure) were measured using a tail-cuff system (BP-2000 Blood Pressure Analysis System, Visitech Systems) in conscious animals. To obtained stable values, mice were preconditioned for three consecutive days (morning and afternoon) and hemodynamic parameters were recorded during the subsequent two days (morning).

### Serum Cholesterol and HSP27 Measurements

An enzymatic assay kit (Wako Pure Chemical Industries, Ltd, Osaka, Japan) was employed to determine serum levels of total cholesterol. Serum HSP27 levels were measured using an ELISA kit specific to human HSP27 (QIA119, Calbiochem, San Diego, CA).

### Preparation of the aorta and evaluation of atherosclerosis

Each aorta was dissected from the ascending to the thoracic segments and the adventitial connective tissue was removed from each segment before it was pinned to a black wax surface using micro-needles and photographed. Thereafter, the aorta was opened longitudinally with the primary incision following the lesser curvature of the arch. To obtain a flat preparation for imaging, a second incision was made along the greater curvature of the arch down to the level of the left subclavian artery. Lipid-rich intraluminal lesions were stained with oil red O and photographed. The *en face* aortic atherosclerotic lesions were analyzed by two observers using Image-Pro software (Media Cybernetics, Silver Spring, MD) to calculate the total and atherosclerotic lesion areas. As described previously the extent of atherosclerosis was expressed as the percentage of surface area of the entire aorta covered by lesions [7].

### Assessment of atherosclerosis in the aortic sinus

The superior half of the heart including the aortic root was embedded in paraffin or frozen in Tissue-Tek O.C.T. media. Serial 4 µm sections of the aortic sinus with valves were cut, beginning at the level where the aortic valve first appears, and stained with hematoxylin and eosin (H&E), Masson's trichrome, oil red O and filipin. Additional tissue sections were used for an apoptosis assay and supplementary immunohistochemical/immunofluorescence labeling. For the quantification of atherosclerotic lesion areas all micrographs were captured with a bright field/fluorescence microscope (Olympus BX60; Olympus America Inc., Center Valley, PA) and analyzed by two observers using Image-Pro software. Lesion area data for each mouse is presented as the mean lesion area of four sections.

### Quantification of cholesterol crystal clefts in atherosclerotic lesions

H&E and Masson's trichrome stained serial sections were employed for assessment of cholesterol crystal deposition in atherosclerotic lesions. Cholesterol clefts were defined as ghost-like needle-shaped spaces that resulted from dissolution of cholesterol crystals during paraffin-embedding and tissue processing. In addition, the necrotic area was defined by the presence of pyknosis, karyorrhexis, or complete absence of nuclei. The cholesterol cleft and necrotic areas were measured using morphometric analysis, as described previously [10].

### Combination of filipin and oil red O staining on the same tissue section

To observe both un-esterified cholesterol and lipoid deposits within the same tissue section, the fluorescent polyene antibiotic filipin and the lipid-soluble dye oil red O were respectively employed according to previously described methodologies [11]. Briefly, a 4 µm-thick cryostat section was incubated in oil red O solution (Sigma; St. Louis, MO) for 1 hour at room temperature. Following washes in PBS, the section was stained with hematoxylin. After repetitive washing in PBS the section was treated with the filipin solution (Sigma) for 2 hours at room temperature. Triplicate washes in PBS were again performed before mounting in 50% glycerol in PBS. Fluorescence was viewed through a D405 nm (blue) and D490 nm (green) barrier filter, respectively. Photomicrographs were taken under the fluorescence exposure followed by bright field using an Olympus BX60 microscope.

### Quantification of macrophage and smooth muscle cell content in atherosclerotic lesions

Macrophage content was assessed using the avidin-biotin-alkaline phosphatase method (Vector Laboratories, Burlingame, California, USA) as described previously [12]. Briefly, tissue sections were pre-incubated with 10% normal horse serum in PBS for 5 minutes followed by a rat anti-mouse macrophage primary antibody (Mac-2; Accurate Chemical and Scientific Corp., Westbury, New York, USA) diluted in PBS 1∶500 at 4°C overnight. After repetitive rinsing with PBS, the sections were incubated with a biotinylated rabbit anti-rat secondary antibody (1∶100, Vector Laboratories, Burlingame, California, USA) for 10 minutes at room temperature. The endogenous peroxidase was quenched with 3% H_2_O_2_ for 10 minutes. Antibody reactivity was detected using an ABC kit (Vector Laboratories, Burlingame, California, USA) and visualized with diaminobenzidine (DAB)/hydrogen peroxidase as chromogenic substrate, resulting in a brown-colored precipitate at the antigen site. As a negative control, tissue sections from each mouse were subjected to the same immunohistochemical protocols but in the absence of the primary antibody. Sections were counterstained with hematoxylin to identify the nucleus, cleared, and mounted. To immunolabel for smooth muscle cell (SMC) content tissue sections were blocked with 10% normal horse serum before being incubated with an mouse anti-α-smooth muscle actin alkaline phosphatase conjugated antibody (αSMA; Sigma, Saint Louis, Missouri, USA) diluted in PBS 1∶60 at 4°C overnight. The visualization of a positive immunoreaction was made possible by the addition of the SIGMA *FAST* Fast Red TR/Naphthol AS-MX Phosphate (4-Chloro-2-methylbenzenediazonium/3-Hydroxy-2-naphthoic acid 2,4-dimethylanilide phosphate, Sigma, St. Louis, MO), which resulted in a red color reaction product. SMC and macrophage areas were quantified using Image-Pro software according to previously described techniques [13].

### Quantification of apoptotic cells in atherosclerotic lesions

Apoptotic cells in atherosclerotic lesions were observed using an *In Situ Apoptosis Detection Kit* (TUNEL, TACS-XL, TA200, R&D Systems, Inc. Minneapolis, MN, USA) and by the detection of activated Caspase 3 using a Cleaved Caspase 3 antibody (Cell Signaling Technology, Inc. Danvers, MA, USA). For TUNEL staining, serial 4 µm sections were incubated with 20 µg/ml proteinase K for 20 minutes at room temperature and washed in PBS. Endogenous peroxidase activity was squelched by incubating with 3% H_2_O_2_ for 10 minutes, washing with PBS and then incubated in 1×TdT Labeling Buffer for 5 minutes at room temperature. Sections were treated with the Labeling Reaction Mix for 1 hour at 37°C and then soaked in 1×TdT Stop Buffer for 5 minutes at room temperature. Following triplicate washes in PBS sections were incubated with an anti-BrdU antibody for 1 hour at 37°C. After triplicate washes sections were covered with a horseradish peroxidase streptavidin solution for 30 minutes at room temperature. The sections were then washed with PBS and treated with 0.03% 3,3′-diaminobenzidine (DAB) containing 0.01% H_2_O_2_ in order to visualize the immunolabeling. Sections were counterstained with hematoxylin to identify the nucleus, cleared and mounted. For negative controls, the Labeling Reaction Mix was omitted. Tissue sections were examined using an Olympus BX60 microscope equipped with a high-resolution digital camera. In each section, the total intimal cell number and TUNEL-positive apoptotic cells within the intima (excluding the necrotic core regions) were manually counted and expressed as the percentage of intimal cells that are TUNEL-positive. For Cleaved Caspase 3 staining, serial 4 µm sections were treated with 0.01 M citric acid buffer (pH 6.0) for 3 minutes in microwave, and then pre-incubated with 10% normal horse serum in PBS for 5 minutes and the sections were incubated with a rabbit anti-Cleaved Caspase 3 antibody diluted in PBS 1∶100 at room temperature for 1 hour. After repetitive rinsing with PBS, the section was incubated with a Texas red goat anti-rabbit secondary antibody (1∶100, Vector Labs) at room temperature for 30 minutes. Following triplicate washes in PBS, tissue sections were incubated with Hoechst 33258 to produce a nuclear signal. Three washes with PBS were again performed before mounting in 50% glycerol in PBS. Photomicrographs were obtained using a fluorescence microscope (Olympus, BX60).

### Combination of immunofluorescence and histochemistry in same tissue section

In order to clearly interpret the morphological relationships amongst various cells and their environments, both immunofluorescence and histochemistry were applied on the same section as described previously [14]. Briefly, in addition of TUNEL and Cleaved Caspase 3 labeling, tissue sections were also label with αSMA/Mac-2 immunolabeling and H&E staining. Tissue sections were pre-incubated with 10% normal horse serum in PBS for 5 minutes followed by a mouse anti-αSMA-FITC (Sigma, Saint Louis, Missouri, USA) diluted in PBS 1∶1000 or a rat anti-mouse Mac-2 (Accurate Chemical and Scientific Corp., Westbury, New York, USA) diluted in PBS 1∶500 at room temperature for 1 hour. After repetitive rinsing with PBS, the section was incubated with a biotinylated rabbit anti-rat secondary antibody (1∶100, Vector Laboratories, Burlingame, California, USA) for 10 minutes at room temperature and assessed as described previously or a Texas red goat anti-rat antibody (1∶100, Vector Laboratories Inc. Burlingame, CA) for 30 minutes at room temperature. Following triplicate washes in PBS tissue sections were incubated with Hoechst 33258 to produce a nuclear signal. Three washes with PBS were again performed before mounting in 50% glycerol in PBS. Photomicrographs were obtained using a fluorescence microscope (Olympus, BX60). Thereafter, the same section was washed with PBS and subjected to hematoxylin staining, and repeat photomicrographs were obtained.

### Quantification of Collagen Content

Picro-sirius red (Sigma) was used to stain for collagen [15], [16]. Co-aligned molecules of Type I collagen display a bright yellow or orange birefringence with polarized microscopy. Image Pro software was used to quantify the intimal collagen content, and the result was expressed as a percentage of the total intimal area.

### Evaluation of aortic stiffness

The effect of HSP27 over-expression on aortic stiffness was assessed using a custom made vessel-stretching device. The vessel dimensions and the force required to stretch a 2 mm aortic ring segment were used to compute the stress induced in the vessel wall and to calculate the stiffness of the thoracic aortic segments. The motorized stage uses a linear voice coil motor (Moticont, CA, USA) mounted on a miniature linear motion ball bearing slide (Edmund Optics Inc., NJ, USA). The real-time position of the motorized stage was recorded using an optical encoder with a resolution of 0.5 µm (MicroE Systems, MA, USA). The motor was controlled with a motion controller (DMC-2143, Galil, CA, USA) fed by the output signal of the optical encoders to execute motion commands. Motion commands are managed using a LabVIEW interface (National Instruments, TX, USA), which controls displacement and speed of the stage. Force was measured using a miniature load cell (model 31 low) with a load range of 0–150 g (Honeywell, MN, USA). Displacement and force measurements were acquired simultaneously using a data acquisition card interfaced with LabVIEW.

The load cell is kept fixed in space while the motorized stage is allowed to move and induce tissue stretch. Hooks are aligned together for mounting of an aortic ring. All samples were kept in Krebs solution at 37°C during stretching experiments. The system was automated to induce a 40% stretch based on the unloaded dimensions of each aortic ring. The tissues were subjected to 12 loading and unloading cycles with a displacement rate of 50 µm/s. The first 11 cycles served to precondition the tissue (40% stretch) and the final cycle was considered as the experimental run. Each aortic ring generated a force-stretch curve. Force data were transformed into engineering stress by dividing the force by the length of the tissue and its thickness. Tissue stiffness was quantified using a MATLAB script (MathWorks, Massachusetts, USA), which calculates the slope of each stress-stretch curve at 30% of stretch.

### Statistical Analysis

All data represent the mean ± standard error of the mean except as specifically stated. Statistical analyses were performed with one-way ANOVA by using SigmaStat 3.5 software. A value of *p*<0.05 was considered statistically significant.

## Results

All mice thrived during these experiments (**Figure 1A**) and body weights were similar between the cohorts, though males were consistently ∼30% heavier than females (**Figure 1B**). The hemodynamic parameters (systolic, diastolic, mean blood pressure and heart beat) of the apoE^−/−^ and apoE^−/−^HSP27^o/e^ mice cohorts at 12 weeks after the beginning of the HFD were similar. Total serum cholesterol levels rose in all groups and were similar in female mice at euthanasia (apoE^−/−^: 1,144±198 *vs.* apoE^−/−^HSP27^o/e^: 1,405±217 mg/dl; *p* = ns). However, in male mice the total serum cholesterol levels at euthanasia were lower in the apoE^−/−^HSP27^o/e^ compared to apoE^−/−^ mice (1,497±304 *vs.* 1,832±267 mg/dl; *p*<0.05). In mice of both sexes serum HSP27 levels rose by more than 16-fold from baseline until completion of a 12 week HFD/euthanasia, however both baseline and final levels were higher in females compared to males (e.g., females: 211±116 to 3,473±1,340 pg/ml, *p* = 0.02; males: 74±23 to 1,204±473 pg/ml, *p* = 0.04) (**Figure 1B**).

### HSP27 over-expression reduces aortic atherosclerotic lesion area

**Figure 2:**
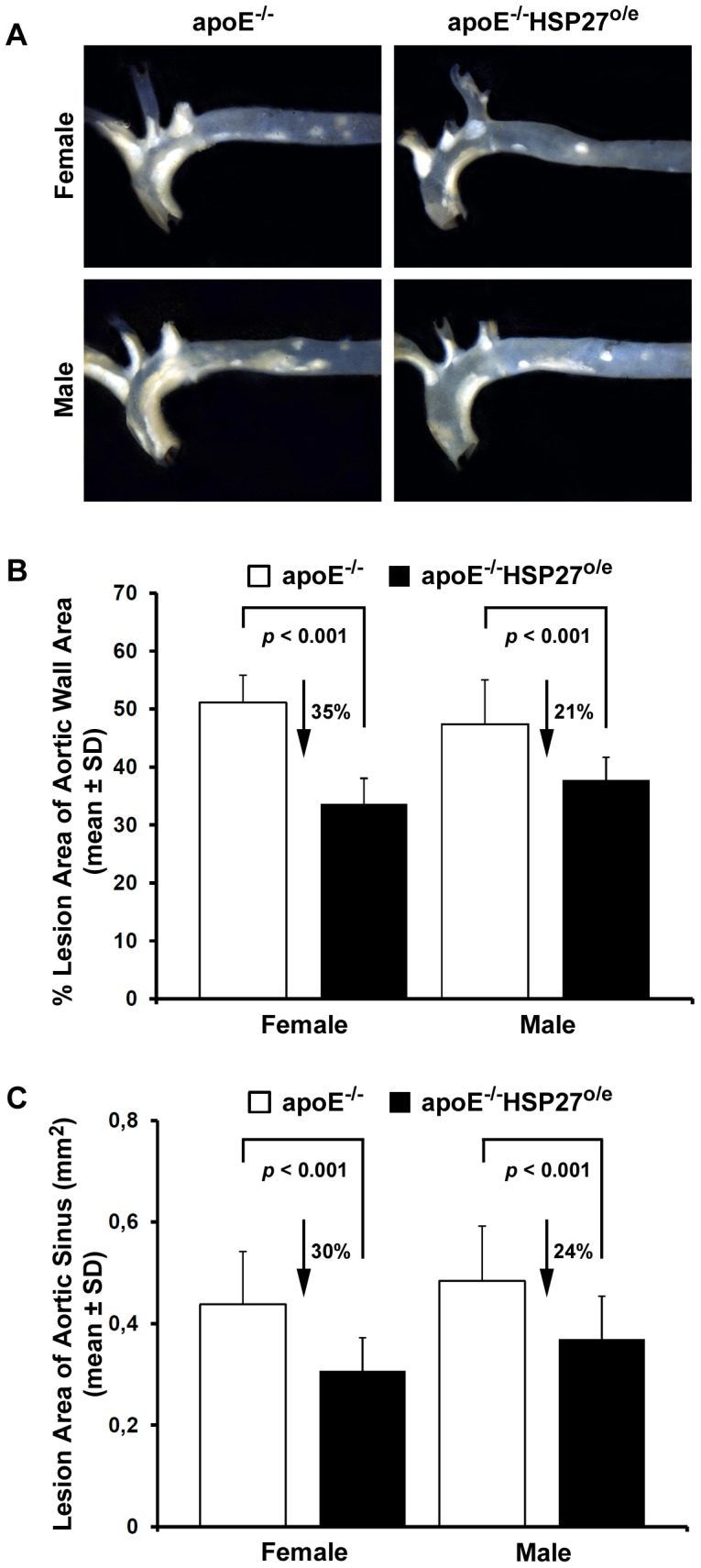
HSP27 over-expression reduces aortic atherosclerotic lesion area in female and male mice. (**A**) The aortic atherosclerotic lesion area was analyzed in apoE^−/−^HSP27^o/e^ mice and compared to apoE^−/−^ littermates. We observed a reduced lesion area in the aortic wall (**B**) and in the aortic sinus (**C**) in apoE^−/−^HSP27^o/e^ compared to apoE^−/−^ mice.

Female apoE^−/−^HSP27^o/e^ mice showed reductions in aortic lesion area of 35% for *en face* and 30% for cross-sectional sinus tissue sections compared to apoE^−/−^ female counterparts (*p*<0.001 for both). Male apoE^−/−^HSP27^o/e^ mice also showed reductions in aortic lesion area of 21% in the *en face* and 24% for cross-sectional sinus sections compared to their apoE^−/−^ male counterparts (*p*<0.001 for both) (**Figure 2**).

### HSP27 over-expression reduces aortic lesion cholesterol content

**Figure 3:**
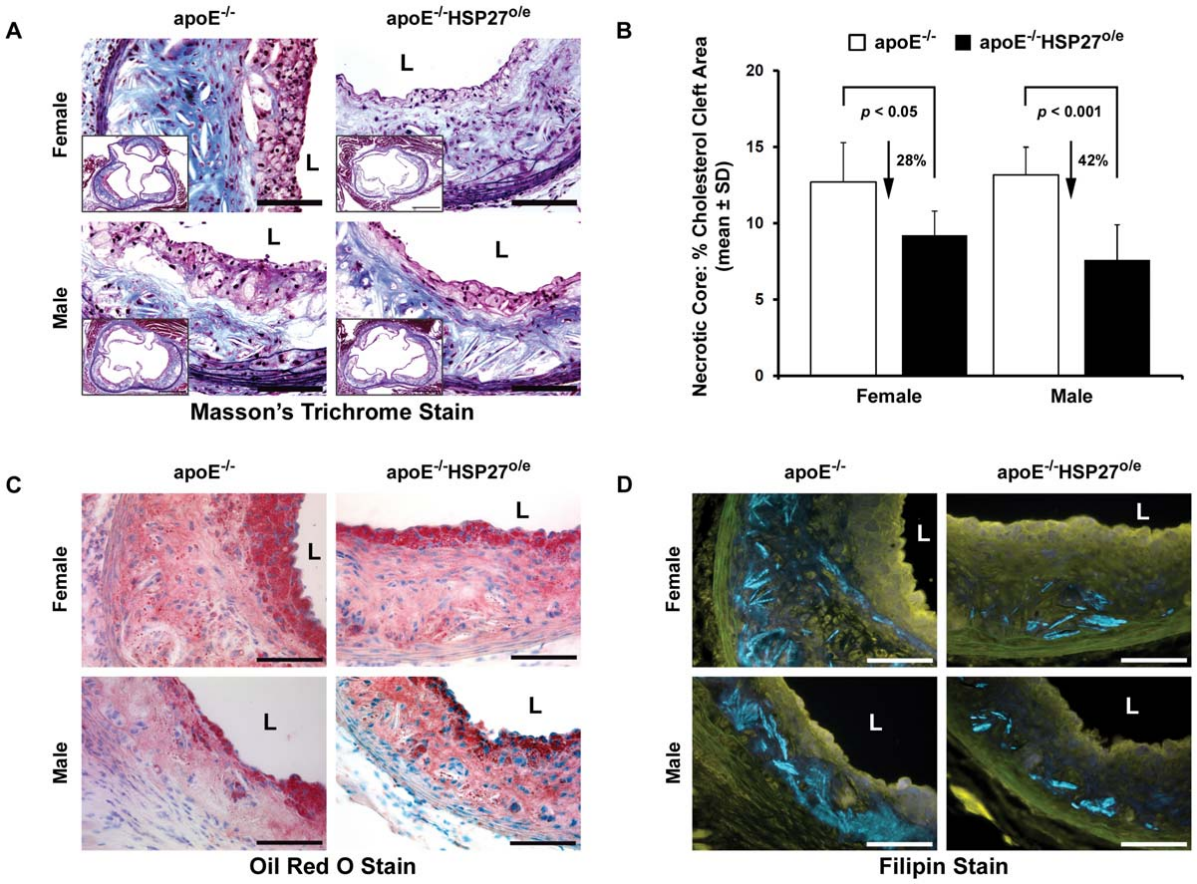
HSP27 over-expression reduces aortic lesion cholesterol content. (**A**–**B**) Cholesterol cleft area is reduced within lesions from apoE^−/−^HSP27^o/e^ *vs.* apoE^−/−^ mice as observed using the Masson's trichrome stain. (**C**) Reduction in arterial wall cholesterol content with over-expression of HSP27 with oil red O staining identifying lipid deposits. (**D**) Attenuated arterial wall unesterified cholesterol content in apoE^−/−^HSP27^o/e^ mice as denoted by fluorescent blue filipin staining. Scale bar = 100 µm (**A**, **C** and **D**) and 500 µm (inserted photo of **A**), L = lumen.

Within the necrotic core of atherosclerotic lesions the acellular area containing cholesterol clefts was reduced in apoE^−/−^HSP27^o/e^ mice by 28% and 42% in females and males compared to their apoE^−/−^ counterparts (*p*<0.05 and *p*<0.001 respectively; **Figure 3A, B**). As well, the intimal lipid and free cholesterol content, as reflected by oil red O (**Figure 3C**) and filipin staining (**Figure 3D**), respectively, were lower in apoE^−/−^HSP27^o/e^ mice compared to apoE^−/−^ mice.

### HSP27 over-expression and lesion macrophage content and remodeling

**Figure 4:**
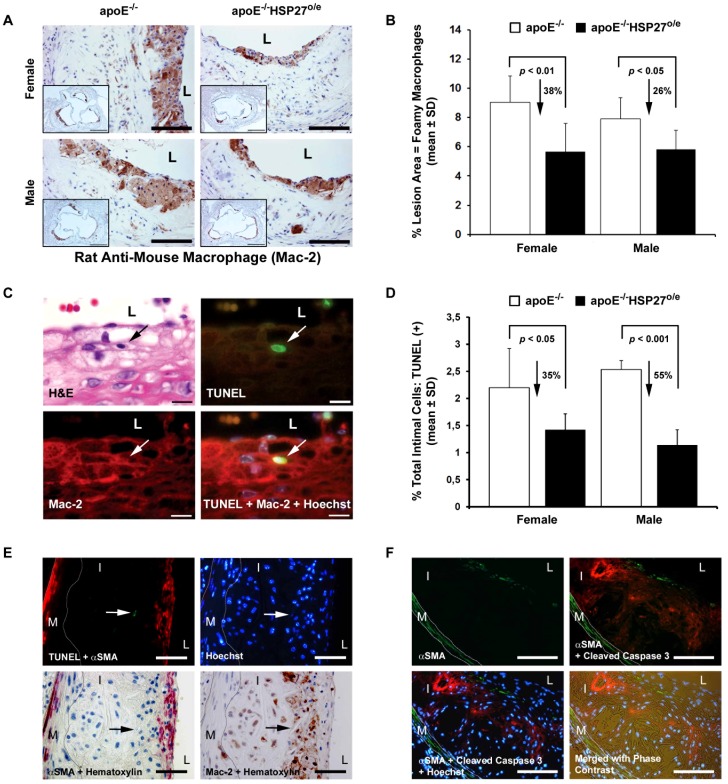
Reduction in arterial wall foamy macrophage content and apoptosis with over-expression of HSP27. (**A**) Macrophages (brown anti-Mac-2 immunolabeling reaction product) localized mainly in layers along the lumenal surface of aortic sinus cross-sections. (**B**) Quantification of macrophage content in aortic sinus cross-sections. (**C**) On a single tissue section stained for histomorphology (hematoxylin and eosin stain; H&E), an apoptotic cell (TUNEL labeling in nucleus; green/arrow) immunolabeled with an anti-Mac-2 macrophage antibody (red immunofluorescence) is identified. (**D**) Quantification of apoptotic cells in aortic sinus cross-sections. (**E, F**) Combination of immunohistochemical and fluorescent TUNEL/Cleaved Caspase 3 staining demonstrating that apoptosis is detected in lesions but do not involve vascular smooth muscle cells. In (**E**), TUNEL (green), αSMA (red), Mac-2 (brown) and Hoechst (blue). In (**F**), cleaved Caspase 3 (red), αSMA (green) and Hoechst (blue). Scale bar = 100 µm (**A, F**), 500 µm (inserted photo of **A**), 10 µm (**C**) and 50 µm (**E**). L = lumen, I = Intima, M = Media, dotted lines delineates the media.

Previously we demonstrated that HSP27 over-expression results in reduced macrophage adherence and migration *in vitro* [7]. As macrophage apoptosis is a prominent characteristic of atherosclerotic plaques (see review, [17]) and HSP27 is a known anti-apoptotic factor [18], we postulated that HSP27 over-expression *in vivo* would result in reduced plaque macrophage content and apoptosis. The aortic sinus cross-sectional area occupied by macrophages was identified using an anti-macrophage antibody (Mac-2) and quantified (**Figure 4A**). Macrophage content in the lesion area was reduced by 38% and 26% in female and male apoE^−/−^ HSP27^o/e^ mice, respectively, when compared to their apoE^−/−^ counterparts (**Figure 4B**, *p*<0.01 & *p*<0.05; respectively). Apoptotic macrophages were identified in lesions using a combination of TUNEL and Mac-2 immunolabeling. TUNEL-positive cells within the nuclei of Mac-2-immunopositive macrophages were found surrounding (**Figure 4C**) as well as within the necrotic core of aortic lesions (data not shown). In the non-necrotic core the percentage of apoptotic cells found in aortic lesions were reduced by approximately 35% and 55% in female and male apoE^−/−^HSP27^o/e^ compared to control sex-matched apoE^−/−^ mice (**Figure 4D**, *p*<0.05 & *p*<0.001; respectively). In order to clarify if vascular smooth muscle cells apoptosis is also present in lesion, we performed double immunohistochemistry/immunofluorescence studies with TUNEL and cleaved Caspase 3 (**Figure 4E, F**). We demonstrate that apoptosis is present in lesions but is primarily found in the necrotic core and did not co-localize with VSMCs.

### HSP27 over-expression and lesion SMC/Collagen content

**Figure 5:**
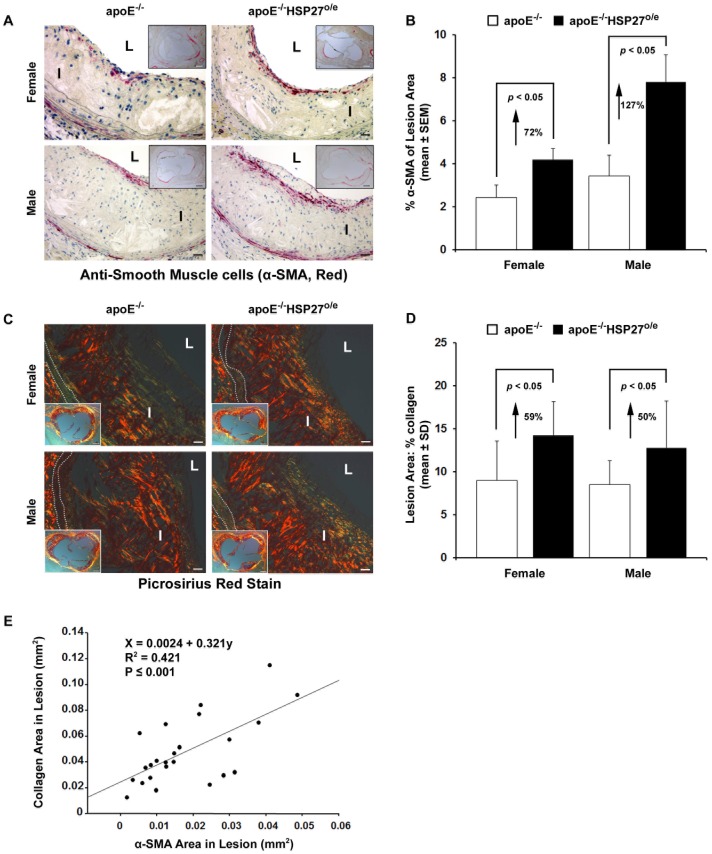
Increased intimal SMC and collagen content with HSP27 over-expression. (**A**) SMC (anti-α-SMA red immunolabeling reaction product) localized mainly in sub-endothelial layers along the luminal surface of aortic sinus cross-sections. (**B**) Quantification of SMC content in aortic sinus cross-sections. (**C**) Intimal collagen content demonstrated by polarized microscopy. Bright yellow or orange birefrigence of collagen, due to co-aligned molecules of Type I collagen. (**D**) Quantification of intimal collagen content. (**E**) Correlation between collagen and SMC content in lesions. Scale bar in (**A**) and (**C**) = 20 µm and 200 µm (insert photo), L = Lumen, I = Intima, dotted lines delineates the media.

The aortic sinus cross-sectional area occupied by SMCs was identified using anti-SMC immunolabeling and quantified (**Figure 5A, B**). SMC content in the lesion area was increased by 72% and 127% in female and male apoE^−/−^ HSP27^o/e^ mice, respectively, when compared to their apoE^−/−^ counterparts (*p*<0.05 for both). Enhanced intimal collagen content is purported to represent a surrogate marker for lesion resilience to plaque rupture [19]. Using polarized microscopy on picro-sirius red stained sections we noted that the abundance of collagen was greater in apoE^−/−^HSP27^o/e^ compared to apoE^−/−^ mice. Indeed, collagen content in the lesion area was increased by 59% and 50% in female and male apoE^−/−^ HSP27^o/e^ mice, respectively, when compared to their apoE^−/−^ counterparts (e.g., females: 14.3±3.9% vs. 9.0±4.6%, *p*<0.05; males: 12.8±5.4% vs. 8.6±2.7%, *p*<0.05; **Figure 5C, D**). Finally, we noted a direct relationship between the abundance of intimal collagen and SMC area in lesions (R^2^ = 0.421, p<0.001; **Figure 5E**).

### HSP27 over-expression increases aortic stiffness

**Figure 6:**
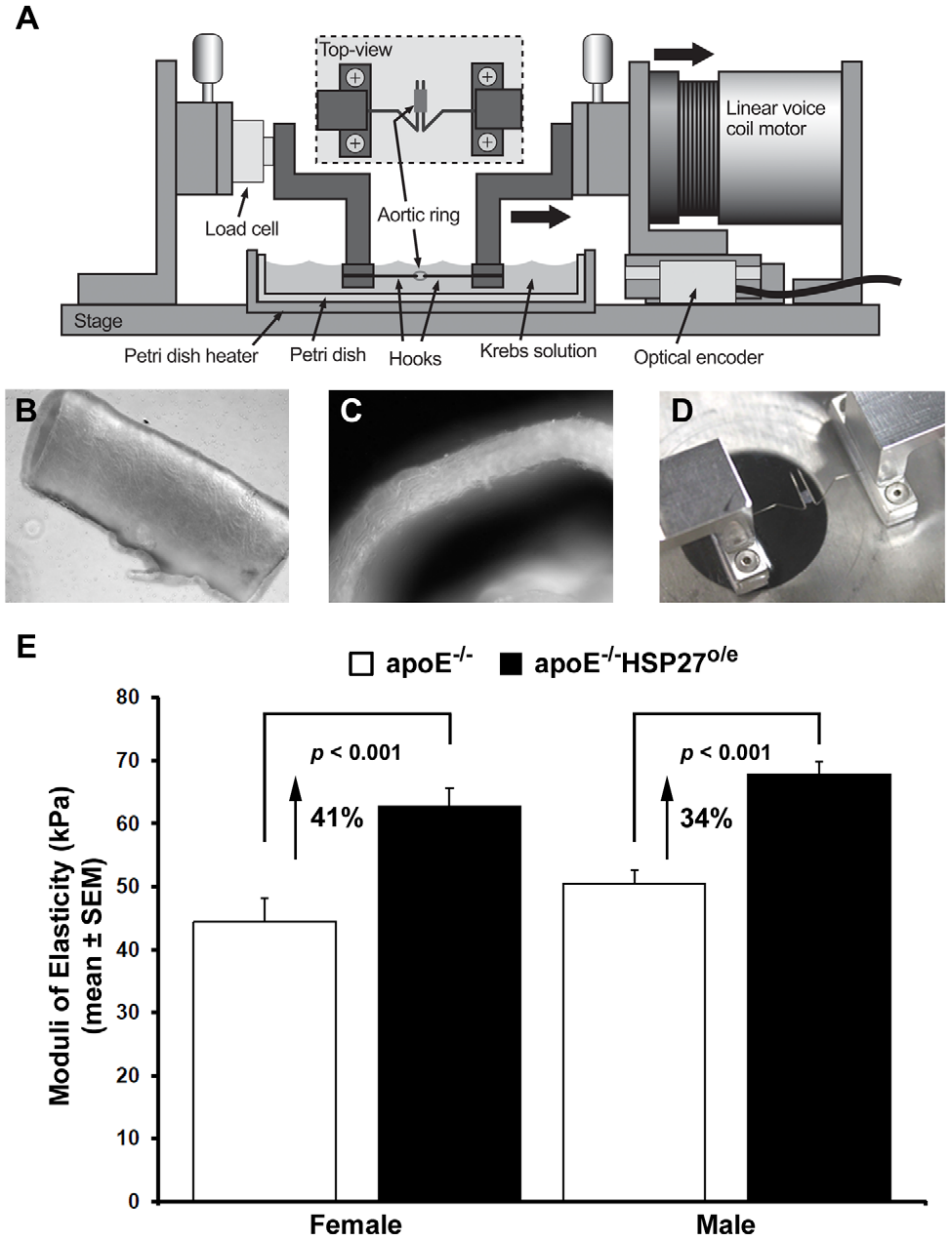
HSP27 over-expression results in increased aortic stiffness. Aortic stiffness measured in apoE^−/−^ and apoE^−/−^HSP27^o/e^ mice fed a high fat diet for 12 weeks. (**A**) The custom made vessel-stretching device is composed of a motorized stage with a linear voice coil motor used to perform loading and unloading cycles. (**B–C**) To determine the stress applied to vessels during stretching, we measured the unloaded vessel area by the microscopic measurement of aortic ring segments length (**B**) and wall thickness (**C**). (**D**) The aortic rings were slipped on two parallel hooks and immersed in a physiological Krebs solution maintained at 37°C. (**E**) Male and female apoE^−/−^HSP27^o/e^ mice showed an increase of aorta stiffness compared to apoE^−/−^ counterparts.

To determine the impact of the enhanced intimal collagen content on the vessel mechanical properties, 2 mm aortic ring segments were precisely measured and preconditioned in preparation for stretching experiments (**Figure 6A–D**). The overall aortic wall thickness of equivalent segments from both murine cohorts was similar (e.g., apoE^−/−^: 128.9±9.3 µm; apoE^−/−^HSP27^o/e^: 124.5±6.8 µm). Changes in the vessel stiffness induced by the over-expression of HSP27 were determined by the calculation of the Young's modulus (Pa) from the stress-strain curves generated during the stretching experiment. The stiffness of the aortic segments in female and male apoE^−/−^ mice were 44.4±3.8 kPa and 50.5±2.2 kPa, respectively and in the same order of magnitude as the stiffness of human tissues that we previously reported [20]. In contrast chronic over-expression of HSP27 resulted in 41% and 34% increases in aortic stiffness (e.g., 62.8±3.0 kPa in females and 67.9±1.9 kPa in males; *p*<0.001 for both groups, **Figure 6E**).

## Discussion

Previously we showed that HSP27 over-expression reduced atherogenesis in female but not male apoE^−/−^HSP27^o/e^ mice fed a HFD for 4 weeks. We also noted a tight inverse correlation between aortic atherosclerotic lesion burden and serum HSP27 levels [7]. In the current study we sought to determine if long-term over-expression of HSP27 attenuates atherogenesis independent of sex. Herein, we found that after 12 weeks on HFD, serum HSP27 levels rose in both male and female apoE^−/−^ HSP27^o/e^ mice. Moreover, both male and female apoE^−/−^ HSP27^o/e^ mice show reduced aortic lesion burdens, with important reductions in plaque cholesterol and macrophage accumulation, as well as increased intimal collagen content and aortic stiffness.

Several important concepts emerge from the current study. First, both sexes of apoE^−/−^HSP27^o/e^ mice showed a more than 16-fold increase in HSP27 serum levels. Female apoE^−/−^HSP27^o/e^ mice had higher baseline and final serum HSP27 levels compared to males. Indeed, male apoE^−/−^HSP27^o/e^ mice had serum HSP27 levels that are similar to those previously observed in female apoE^−/−^HSP27^o/e^ mice after ingesting a HFD for just 4 weeks [7]. These higher serum levels of HSP27 in the female mice correlate with important earlier observations from our laboratory. Previously we demonstrated *in vitro* that estrogens promote the extracellular release of HSP27 from macrophages, and that *in vivo* HSP27 over-expression in ovariectomized mice requires estrogen replacement therapy to restore both the rise in serum HSP27 levels and atheroprotection that normally occurs when these mice are placed on a HFD [7], [8]. Presumably the rise in serum HSP27 levels seen in the male mice from the current study is the result of prolonged exposure to the metabolic influences of an atherogenic HFD – as before we noted *in vitro* that macrophages treated with acLDL also secreted HSP27 [7]. Detecting HSP27 levels in the serum is a recent phenomenon, as HSPs are traditionally considered as intra-cellular chaperone proteins – yet an expanding number of HSPs are now identified in the extracellular space and implicated in macrophage signaling events (see review, [21]). While our understanding of the mechanisms by which HSPs exit cells is incomplete, we demonstrated that the release of HSP27 from macrophages occurs via an exosomal pathway [8].

Second, it is intriguing to consider why HSP27 over-expression is associated with a reduction in plaque cholesterol cleft area and lipid content. In a previous study, we showed that extracellular HSP27 interacts with Scavenger Receptor-A (SR-A) and competitively inhibits the uptake of acLDL *in vitro* by 41% [7]. Hence, we have reason to believe that HSP27 interferes with cholesterol uptake by macrophages, and our recent studies suggests that HSP27 reduces SR-A expression and the ability of cells to take-up cholesterol [22].

Third, in the current study we observed an important reduction in the macrophage content of lesions. Previously we noted *in vitro* that adhesion and migration is attenuated in macrophages derived from apoE^−/−^HSP27^o/e^ compared to apoE^−/−^ mice [7]. Yet, macrophage abundance in lesions is not only determined by the number of macrophages that enter lesions – but also by the survival and exit of macrophages from the plaque. Although HSP27 is known to have anti-apoptotic effects, despite this stabilizing effect on macrophage survival, there are still fewer macrophages in lesions of HSP27 over-expressing mice. At this stage, we are unable to comment on the dynamics of macrophage transit from lesions, but we now know that HSP27 dramatically upregulates GM-CSF, a cytokine known to play an important role in mononuclear cell trafficking and re-entry into the circulation [3]. Hence, it is attractive to posit that HSP27 has a stabilizing effect on vessel wall macrophages, not only reducing their ability to sequester cholesterol as foam cells, but also reducing their adherence and entry into lesion, as well as promoting their survival and potential egress from the plaque.

Fourth, we note that the lesions of HSP27 over-expressing mice undergo a multi-faceted histological remodeling process that is consistent with a more resilient lesion. The characteristics of this lesion remodeling include: i) reduced macrophage content, ii) reduced apoptotic cells in the non-necrotic areas of lesions, iii) increased intimal SMC and collagen content, and iv) increased aortic rigidity. Although we are unable to ascertain a direct connection between increased SMC and collagen content with the vessel wall stiffness, these biological changes in the vessel wall are certainly consistent with the altered mechanical properties that we measured. It is interesting to note that, while intimal collagen content was higher for both the female and male apoE^−/−^HSP27^o/e^ compared to the apoE^−/−^ mice, the difference was greater for males. One possible explanation for the less impressive increase in collagen content for the female apoE^−/−^HPS27^o/e^ mice may be the known negative effect that estrogens have on the expression of collagen [19]. Certainly, collagen expression is recognized as an important part of complex human coronary artery lesions [23]. The abundance of collagen in lesions reflects the balance between production and destruction. Hence it is attractive to postulate that the higher collagen content of the apoE^−/−^HSP27^o/e^ murine lesions reflects less destruction of collagen – a possibility that might be predicted from our previous observations that HSP27 facilitates a shift towards an anti-inflammatory vascular milieu (e.g., increase in IL-10 with a decrease in IL-1β) [3], [7]. Taken together, these features of vessel wall remodeling are important surrogate end-points for plaque resilience and support the contention that HSP27 promotes favorable vascular remodeling.

**Figure 7:**
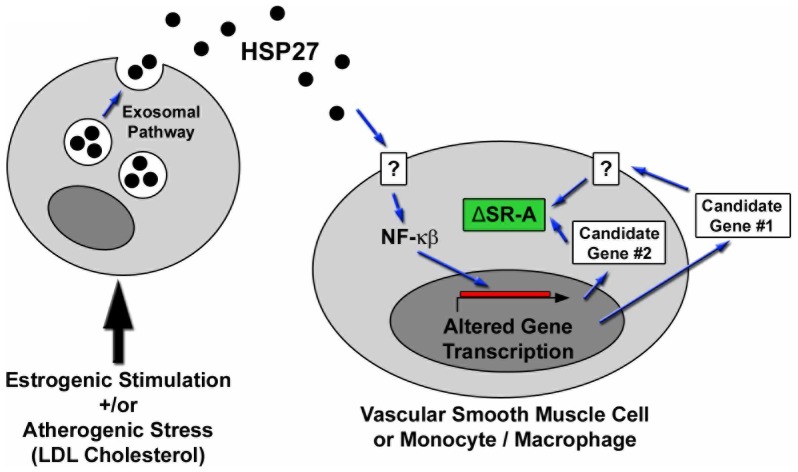
HSP27 release and cell-signaling. In response to estrogenic stimulation or to atherogenic stress induced by elevated serum cholesterol levels, HSP27 is released into the extracellular space via an exosomal pathway. Once available in the extracellular space, HSP27 may alter gene transcription in target cells (e.g., vascular smooth muscle cells or monocyte/macrophages). Shown here is HSP27-induced activation of NF-κB, resulting in candidate genes expression and the altering of scavenger receptor-A (SR-A) expression, thereby reducing cholesterol uptake and foam cell formation. The search for a putative HSP27 cell surface receptor is ongoing.

Finally, it is interesting to note lower total serum cholesterol levels in the male apoE^−/−^HSP27^o/e^ *vs* apoE^−/−^ mice. While this difference in serum cholesterol levels is not found in the female mice, total serum cholesterol levels are not as high in the female mice compared to the males. Although the precise mechanistic explanation for this difference in serum cholesterol levels remains unclear, we recently identified that HSP27 plays a key role in NF-κB signaling – including the induction of a number of important factors that may influence cholesterol levels [3]. Hence, we speculate that the reduction in serum cholesterol levels observed in the male apoE^−/−^HSP27^o/e^ mice may be due to downstream transcriptional effects (**FIGURE 7**). Certainly, longer duration experiments are needed to ascertain if these differences in serum cholesterol levels become more striking over time and remain isolated to males.

Taken together, these data indicate that chronic HSP27 over-expression results in atheroprotection that is independent of sex. Moreover, HSP27-mediated atheroprotection occurs in association with remarkable reductions of plaque lipid and macrophage abundance plus an increase in intimal collagen content and vessel/plaques stiffness. While our studies are too short to be conclusive, we surmise that the enhanced vascular remodeling seen with HSP27 over-expression may offer an important advantage by enhancing the resilience of lesions and potentially preventing plaque rupture. Preliminary therapeutic studies utilizing a recombinant form of HSP27 are ongoing.
